# Resonant Raman Spectroscopy of Carotenoids in Aging of Extra Virgin Olive Oil

**DOI:** 10.3390/s23177621

**Published:** 2023-09-02

**Authors:** Edward Curran Eggertson, Francesca Venturini

**Affiliations:** Institute of Applied Mathematics and Physics, Zurich University of Applied Sciences, Technikumstrasse 9, 8401 Winterthur, Switzerland

**Keywords:** resonant raman spectroscopy, raman spectroscopy, fluorescence spectroscopy, carotenoids, olive oil

## Abstract

This work uses resonant Raman spectroscopy (RRS) to investigate changes in carotenoid concentration in extra virgin olive oil (EVOO) as it oxidizes under accelerated thermal aging. Carotenoids are nutritious antioxidants and biomarkers that represent the general quality of olive oil. HPLC is the conventional method used to determine the concentration of carotenoids, but it is expensive, time-consuming, and requires sample handling. A simple optical technique for estimating carotenoid concentration in extra virgin olive oil is, therefore, desirable. This work shows that the normally weak carotenoid signal is strongly amplified when using the resonant Raman technique. The aging and oxidation of EVOO decreases the Raman intensities associated with carotenoids and increases the fluorescence and Raman intensities associated with fatty acids. From these quantities, two Raman intensity ratios are presented as indicators of the effects of aging.

## 1. Introduction

Olive oil is prized for its nutritional value and numerous health benefits, including its antioxidant content. The highest quality olive oil—Extra Virgin Olive Oil (EVOO)—is protected through classification methods and regulations. Classification is currently an onerous process that relies on several chemometric and optical measurements, so a robust optical classification method that is faster and cheaper than the current methods is highly desired. Carotenoids are highly concentrated in olive oil and act as antioxidants, delaying aging processes both in the oil and in the consumer [1]. Furthermore, carotenoids can be used as a biomarker such that a low carotenoid concentration indicates that the olive oil is old, has been exposed to poor storing conditions, has been heated during extraction, or has been adulterated with cheaper oils.

Raman spectroscopy (RS) is an optical method that has been applied to the quality control of EVOO since Baeten in 1996 [2], focusing on the characterization of fatty acids and, more recently, carotenoids. The main disadvantage of this technique is the weak Raman cross-sections, especially when compared to a strong, broadband fluorescent background signal. This work proposes the use of resonant Raman spectroscopy (RRS) to significantly increase the Raman cross-section of carotenoids.

In the present study, commercial EVOO samples underwent accelerated aging in an oven to induce the thermal oxidation of carotenoids. The resonant Raman spectra of the samples were measured, and the spectra were processed to remove the influence of confounding signals, which were then quantified via curve fitting for further analysis. The goal of this study is to investigate the dependence of the EVOO Raman spectra on accelerated thermal aging and identify useful indicators in the quality control of EVOO.

## 2. Background

### 2.1. Oxidation and Lipolysis in Olive Oil

Olive oil is composed mainly of triglycerides, which comprise a glycerol backbone connected to three fatty acids (FAs). The FA profile of an olive oil is indicative of the cultivar, the maturity of the fruit, the climate, etc. An oil’s FA profile is traditionally determined through gas chromatography [3]. However, RS has also been used with mixed results for at least classifying the level of unsaturation of the FAs [1,4].

Lipolysis and—more importantly—oxidation are the two main processes contributing to the decreasing quality of olive oils with age [5]. Lipolysis concerns the decomposition of triglycerides into free fatty acids (FFAs). FFAs increase the acidity of olive oil, and a low FFA content is required to qualify an oil as EVOO. FFAs are given as a percentage of total FAs, with EVOOs typically ranging from 0.5–0.8%. Oxidation concerns the decomposition of individual fatty acids when exposed to reactive oxygen species. Oxidation products give an unpleasant flavor and odor and possibly adversely affect the nutritional value of the oil. FA oxidation is hindered by the olive oil’s natural antioxidants (e.g., carotenoids) that consume these reactive oxygen species [5]. Heat is a catalytic factor in oxidation, so aging can be accelerated through heating, hence the prominence of cold extraction processes in EVOO production.

### 2.2. Quality Assessment of EVOO

The quality assessment for EVOO is defined through a series of parameters with defined upper limits [6,7]:

- FFA content, which is driven by lipolysis;
- Peroxide content, which is driven by FA oxidation, thereby indicating the extent of oxidation in the oil. Peroxides also promote further oxidation and can decompose into aldehydes and ketones, which have a substantial negative effect on oil flavor;
- Oleic acid content, which allows for differentiation between olive oil and other edible oils;
- Three optical parameters concern UV absorptivity, which is related to the concentration of primary and secondary oxidation components, i.e., peroxides and aldehydes/ketones [8].

Like peroxide concentration or UV absorptivity, low carotenoid concentration also indicates oxidation of the oil. However, directly measuring carotenoid content has the advantage of evaluating the value of the oil as an in vivo antioxidant. Carotenoid concentration is not currently part of the regulations, in part because it is measured chemometrically via high-performance liquid chromatography (HPLC), which is a burdensome process compared to optical methods. An optical method to estimate carotenoid concentration would be an attractive supplement to the existing regulations.

### 2.3. Raman Spectroscopy

Raman spectroscopy (RS) is an optical technique for measuring the chemical fingerprint of a sample. When excited with a coherent light source, a sample emits inelastically scattered light, with a spectrum comprising several peaks and a constant Raman shift (measured in cm${}^{-1}$) relative to the source. The peaks of Raman spectra are highly conserved and distinct when compared to those from infrared spectroscopy (IRS). However, the weak cross-section of Raman scattering is a significant challenge to practical RS. Several RS methods have been used in the literature to investigate the Raman emissions of carotenoids and/or olive oil.

Surface-enhanced RS (SERS) was used in conjunction with wavelet analysis to extract the Raman signal from the very strong fluorescence background in the Raman window of a 633 nm excitation [9]. Other studies have cited 633 nm as the worst excitation for fluorescence suppression [10]. Fourier transform (FT) RS is a popular technique, as it uses the same equipment as FT-IRS absorptive spectroscopy yet yields complementary information about the sample. Near-infrared (NIR) RS is a similar method, but it uses a diffractive spectrometer instead of an interferometer. Importantly, the 1064 nm excitation of FT-RS and 785 cm${}^{-1}$ excitation of NIR-RS yield Raman windows above the influence of fluorescence but also very low spontaneous carotenoid Raman cross-sections. FT-RS [2,11,12,13] and NIR-RS [10,14,15,16,17,18], therefore focusing primarily on estimating the FA profile and detecting olive oil adulteration. However, to measure carotenoids, resonant RS is preferred.

#### 2.3.1. Resonant Raman Spectroscopy

The cross-section of a Raman emitter—and, therefore, the intensity of the Raman signal—depends on the excitation wavelength. As with inelastic scattering, a spontaneous (unenhanced) Raman cross-section tends to decrease sharply with increasing wavelength ($\propto \lambda^{-4}$) [19]. In addition, carotenoids experience the resonant Raman effect, where the Raman cross-section deviates from the spontaneous Raman cross-section at certain excitation wavelengths, enhanced by up to a factor of $10^{5}$ [19]. This yields the method of resonant Raman spectroscopy (RRS).

The enhancement, denoted as f(λ), is defined by the resonant Raman excitation profile (RREP) of a Raman emitter. The theoretical RREPs for carotenoid species lycopene [20] and lutein [21]—each found in an acetone solution—are available for carotenoids, yielding a resonant range of 460–516 nm. However, the author claims that the RREPs are dependent on the solvent [21] and may differ in oil. Lu offers an absorbance spectrum for β-carotene in acetone, intended to closely correlate to an RREP [22]. These experimentally derived data suggest a strong preference for 488 nm over other RRS excitations, but an in situ RREP in EVOO would be a useful tool for the experimental design of RS for olive oils.

The potential of RRS to detect carotenoids in EVOO has attracted increasing attention in recent years, using 488 nm [23], 514 nm [4,24] and 532 nm [25,26,27] excitations; hence, this is why the technique is also sometimes referred to as visible (VIS) Raman spectroscopy [24].

#### 2.3.2. Raman Spectroscopy for Olive Oil

All the peaks in the Raman spectra of EVOO are attributable to either carotenoids or FAs. In the visible range, resonant enhancement causes carotenoid peaks to dominate over FA peaks. In the near-infrared, carotenoids are out of their resonant range, and the FA peaks become dominant due to a higher spontaneous cross-section, their own weak resonances, and the relative lack of fluorescence.

The peaks associated with FAs within the current Raman window are found at 1270, 1305, 1440, 1655, and 1750 cm${}^{-1}$ [1]. The intensity of these peaks increases slightly with increasing FFA content, so the lipolysis of EVOOs has been investigated with RS, where the level of FFAs is evaluated [10,28]. However, El-Abassy et al. actually found that the larger changes (decreasing) in the peaks associated with carotenoids were actually a better predictor of FFA content than the smaller changes (increasing) in the FA peaks themselves [28].

In order to obtain a clean Raman signal, the significant contribution of fluorescence (attributed primarily to chlorophyll [9]) must be suppressed (or isolated). An investigation by Wei et al. [29] evaluated several suppression techniques, including fast-gating, anti-Stokes scattering, IR excitation, SERS, RRS, and background subtraction; the latter two methods were used in the current study. Many other studies utilize NIR-RS or FT-RS with IR excitation to avoid the influence of chlorophyll fluorescence, which emits most strongly at 670 nm.

## 3. Materials and Methods

### 3.1. Experimental Setup

**Figure 1:**
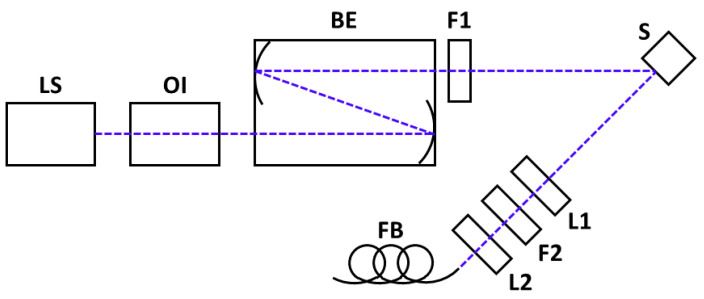
The experimental setup is shown, comprising a light source (LS), optical isolator (OI), beam expander (BE), cleanup filter (F1), the sample (S), collimating lens (L1), and a notch filter (F2) and lens (L2) for focusing the fiber bundle (FB).

The Raman setup is based on an oblique backscattering geometry (Figure 1), which decreases alignment sensitivity and increases the signal-to-noise ratio (SNR) when compared to a typical 90° geometry. Excitation is provided by a 32 mW 488 nm laser diode (LS; 0488L-21A, Integrated Optics, Vilnius, Lithuania). Back-reflection is prevented by an optical isolator (OI; IO-3-488-HP, ThorLabs, Newton, NJ, USA). Photo-oxidation is avoided by decreasing irradiance with a reflective beam expander (BE; BE06R/M, ThorLabs, Newton, NJ, USA). Side mode suppression is achieved with a cleanup filter (F1, Semrock HC Laser Clean-up MaxLine 488/1.9, IDEX, Rochester, NY, USA). A pair of lenses are used for collimation (L1; *f* = 10 cm) and focusing (L2; *f* = 2 cm) of the Raman emissions on the fiber bundle (FB). Scattered laser light was removed by a notch filter (F2; ZT488TopNotch, Chroma, Bellows Falls, VT, USA), between L1 and L2. The FB transmits the Raman emissions to a spectrometer (IsoPlane 160, Princeton Instruments, Trenton, NJ, USA) using 1200 g/mm grating and an entry slit width of 25μm, coupled to a CCD sensor (ProEM 1024 EMCCD, Princeton Instruments, Trenton, NJ, USA). The fluorescence excitation-emission matrix (EEM) was measured using a Cary Eclipse Fluorescence Spectrometer (Agilent Technologies, Santa Clara, CA, USA) at a constant temperature of 22 ${}^{{}^{\circ}}$C on undiluted samples.

### 3.2. Sample Preparation

Five commercially available EVOOs were selected, being heterogeneous in origin (within the EU) and cost, and these were identified as Oil A through to Oil E. The samples were thermally aged at 60 °C to induce accelerated oxidation, which loosely follows the Schaal oven test, such that each day of aging is equivalent to a month of aging at ambient conditions [5], although smaller sample volumes were used (5 mL vs. 120 mL). Six samples were extracted from each freshly opened bottle, one acting as an unaged reference and the others aged to 4, 9, 18, 27, and 53 days, yielding 30 total samples (example reference B-53 d). The samples were aged in closed glass containers with minimal headspace and were transferred to 3.5 mL quartz cuvettes for testing to avoid the fluorescence that is typical of glass containers. Quartz’s own Raman signal is outside of the relevant Raman window for this work.

### 3.3. Signal Analysis

The raw signal composition includes the Raman signal and a baseline, where the baseline comprises background radiation (mostly fluorescence) and the sensor signal (dark signal and readout bias). Signal processing was conducted as follows:

1. Correct for notch filter transmission to remove the artefactual interference peaks that have the same apparent bandwidth as a Raman peak;
2. Remove the readout bias and the dark signal from the spectrum baseline. The remaining baseline (95+% of the initial baseline) is primarily fluorescence;
3. Fit a model to the spectrum for the simultaneous quantity extraction and isolation of the Raman and fluorescence signals.

**Figure 2:**
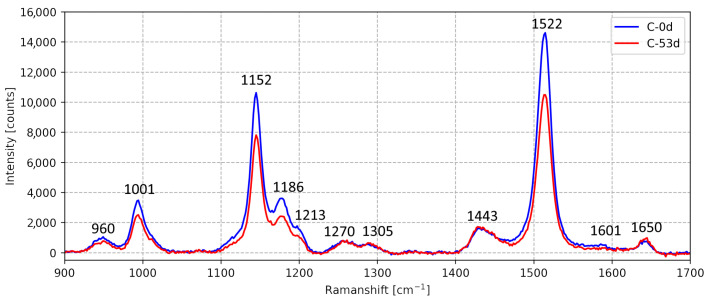
Raman spectra of Oil C (fresh) (C-0d) and the 53-day-aged oil (C-53d) show the effect of aging. Each significant peak’s Raman shift is annotated. The region of interest is the 900–1700 cm${}^{-1}$ carotenoid Raman window.

The removal of the fluorescent background from the Raman signal was enabled by the dissimilar bandwidths of these features, with ($FWHM_{Raman}∼$30 cm${}^{-1}$ vs. $FWHM_{fluor}∼$500 cm${}^{-1}$). The model comprises a polynomial function to fit to the baseline and several Lorentzian functions, each representing a prominent Raman peak. While over 12 Raman peaks are identifiable in the Raman spectra in the domain of interest (Figure 2), only 6 Lorentzian elements, representing the 6 most prominent peaks (listed in Table 1), were included in the model as this increases the stability of the fit, and the intensities of the less prominent peaks are not useful for further analysis. The intensities of the 6 fitted Raman peaks and average baseline (fluorescence) intensity were tabulated for all 30 samples.

Peak broadening of the Raman signal can occur if the spectral resolution of the spectrometer and the line width of the laser are not significantly narrower than the Raman peaks. The line width of the single-mode laser diode was <0.01 nm (0.4 cm${}^{-1}$). The spectral resolution of the spectroscope was determined using the very narrow 2330 cm${}^{-1}$ Raman peak of $N_{2}$, as 0.17 nm (7.0 cm${}^{-1}$). Both values are much less than the typical $FWHM_{Raman}$, indicating a low degree of instrument-specific peak broadening and, therefore, a negligible effect on the measured peak height.

## 4. Results

### 4.1. Fluorescence

**Figure 3:**
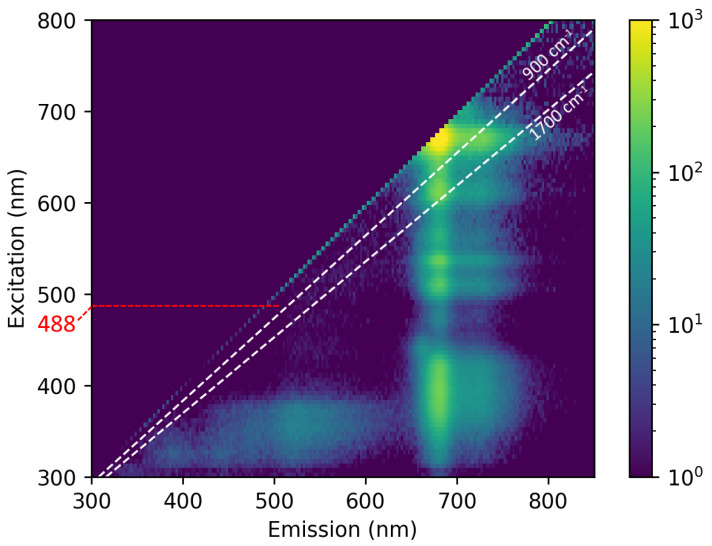
An excitation–emission matrix of EVOO (Oil A) is presented with a logarithmic color scale. The white lines represent the bounds of the carotenoid Raman window of 900–1700 cm${}^{-1}$. The 488 nm excitation yields relatively low background fluorescence in this window.

The experimental design, namely source selection, of the RSS of EVOO should maximize the Raman signal while minimizing fluorescence by balancing the RREP (carotenoid signal) and the fluorescence EEM. Figure 3 shows the EEM of Oil A, as measured using a fluorescence spectrometer. The most dominant features are the diagonal of Rayleigh-scattered light, the strong fluorescence of chlorophyll emitting at ∼670 nm, and the moderate broadband fluorescence from UV excitation (<400 nm).

The Raman window is depicted by the white lines and experiences low fluorescence for 380–570 nm excitation. This aligns well with the assumed carotenoid RREP and is centered on the 488 nm source used. However, the fluorescence in the Raman window from the 440 nm excitation is about ¼ as strong as the fluorescence from the 488 nm excitation. This excitation can be achieved hypothetically by using a 442 nm HeCd laser, which is sometimes used for RS. Furthermore, according to [22], the Raman cross-section of carotenoids at 450 nm may be even higher than at 488 nm. The use of a HeCd laser (or an alternative) for this application may further suppress fluorescence and be useful for future studies on carotenoids.

### 4.2. Aging

**Figure 4:**
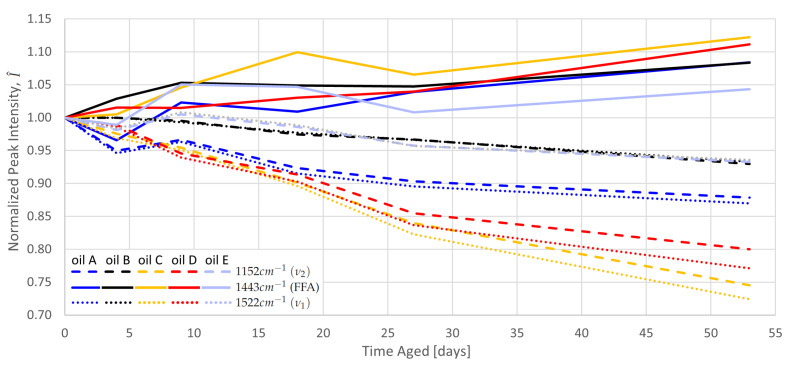
Aging behavior of the normalized Raman peak magnitudes (relative Raman intensity, $\hat{I}$) for each EVOO.

For each sample, seven primary quantities were extracted from the the measurement: the average baseline in the Raman window (representing fluorescence intensity) and the intensity of the six fitted Lorentzian peaks. Figure 4 shows how three of the six peak intensities of each oil developed with aging. As the peaks have very different magnitudes due to the heterogeneity of the oils, they are normalized to their un-aged intensity value for better comparison, (${\hat{I}}_{xd}=I_{xd}/I_{0d}$). The trends of the unplotted peaks are congruent with those of the plotted carotenoid peaks. As expected, the carotenoid peak intensities decrease (oxidation) while the FA peak increases (lipolysis). When normalized to their $I_{0d}$ value, the rate of change of all peaks, $\left|d\hat{I}/dt\right|$, is similar for a given oil. However, this quantity is not conserved between the oils, as some (e.g., C) are more sensitive to the aging process than others (e.g., B).

**Figure 5:**
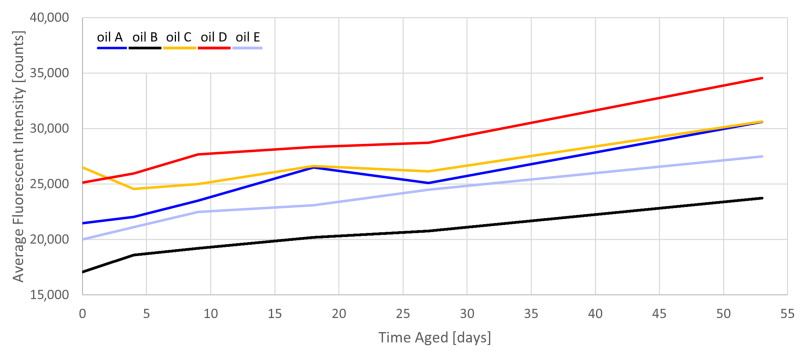
Aging behavior of the average fluorescent baseline intensity (unnormalized).

Figure 5 demonstrates the increase in fluorescence with age for all EVOOs. While the oils have a different starting fluorescent intensity, the growth rate is consistent for all oils. Although most sources in the literature cite chlorophyll as the primary source of fluorescence [9,30], this does not explain the increase in fluorescence since chlorophyll cannot accumulate with age. Rather, chlorophyll is responsible for the very strong fluorescence at longer wavelengths (∼670 nm) [31], which does not affect the Raman spectra of this study. The relatively weaker fluorescence in the Raman window is loosely attributed to lipolytic and oxidative products, which does explain the fluorescence increase (consistently 25–30% by 53 d).

## 5. Discussion

Figure 4 shows that the change in any individual peak intensity can be an indication of aging in any oil. However, some measurements (e.g., $I_{A-4d}$) show an unexpected drop in intensity. The intensity of a Raman signal (*I*) is proportional to the laser intensity $I_{0}$, emitter cross-section (σ), emitter concentration (*D*), and collector efficiency (Ω). It would be appealing to set D∝I for a calibrated device, holding the other variables constant. However, these other variables rely too strongly on the test hardware and methodology. McCreery describes Raman intensity as “very difficult” to analyze in an absolute manner and better for detecting “day to day instrument performance” and “hardware and alignment problems” than changes in concentration [19]. Therefore, the degree of aging or carotenoid concentration should not be predicted directly from the *I* or $\hat{I}$ of a single peak.

An ideal indicator would allow for the evaluation of time aged (or accumulated oxidation) from a single spectrum. Such an indicator, Γ, plotted over time must exhibit four important, progressively restrictive criteria:

1. The indicator is independent of $I_{0}$, Ω, or other factors that aren’t *D*;
2. The indicator exhibits a steep slope with age, which increases its dynamic range and sensitivity;
3. The decay time constant is consistent between different EVOOs, such that the indicator normalized to the initial value, $\hat{\Gamma }=\Gamma /\Gamma_{0d}$, is equal for all EVOOs;
4. The indicator’s initial value, $\Gamma_{0d}$, is equal between all EVOOs or proportional to some other initial measurement of quality, which allows for direct evaluation without normalization.

Criterion 1 is achieved by using the ratios of the quantities derived from a single spectrum, or Raman Intensity Ratios (RIRs) [25]. For example, the intensity ratio of $\Gamma =\nu_{1}:\nu_{2}$ has been used to identify the dominant species of carotenoids in a solution (e.g., lutein vs. β-carotene) [32]. This particular Γ is unlikely to be beneficial in this scope, as the two peaks scale very closely with each other in all measurements (Figure 4). RIRs are immune to most errant changes in spectral intensity, as these should affect both the numerator and denominator equally.

Criterion 2 is achieved by using RIR input quantities that (a) have high rates of change and (b) have rates of change that are opposite to each other. For example, the criterion can be satisfied by using a decreasing quantity (e.g., carotenoid peak intensity) as the numerator and an increasing quantity (e.g., fluorescence or FA peak intensity) as the denominator.

**Figure 6:**
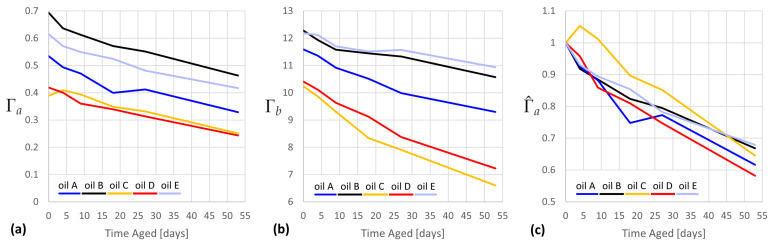
Two possibilities for absolute indicators: (**a**) $\Gamma_{a}=\nu_{2}:fluor$ (**b**) $\Gamma_{b}=\nu_{1}:\nu_{1444}$; (**c**) ${\hat{\Gamma }}_{a}$ ($\Gamma_{a}$ normalized to $\Gamma_{0d}$).

From these criteria, we evaluate two possible indicators, $\Gamma_{a}$ and $\Gamma_{b}$, each presented in Figure 6, plotted over time for each oil. Each quantity uses a decreasing intensity ($\nu_{2}$ or $\nu_{1}$, resp.) in the numerator and an increasing intensity in the denominator (average fluorescence or the FA peak at 1444 cm${}^{-1}$, resp.). For each, the dynamic range is good over the aging range.

In order to satisfy criterion 3, the exponential decay of Γ should have the same time constant for all EVOOs. This is true if the normalized indicator becomes approximately colinear for all oils, which is demonstrated in Figure 6c for ${\hat{\Gamma }}_{a}$ but does not hold for ${\hat{\Gamma }}_{b}$ (not pictured). $\Gamma_{a}$, therefore, satisfies criterion 3.

In order to satisfy criterion 4, $\Gamma_{a}$ would either need to be colinear for all oils when unnormalized (untrue according to Figure 6a) or would have to have a $\Gamma_{0d}$ for each oil that is proportional to the assumed amount of total accumulated oxidation during manufacture and transport. One would, therefore, expect that the higher-quality oils (indicated by higher cost per liter) would have less accumulated oxidation and, therefore, have higher $\Gamma_{a,0d}$. However, the opposite is true, with EVOOs B and E being many times cheaper than EVOOs C and D. $\Gamma_{a}$, therefore, does not appear to satisfy criterion 4, though this cannot be excluded without a comparison to the chemometric analysis of the samples.

Directly using ${\hat{\Gamma }}_{a}$ can be a very good predictor for the time aged. For example, ${\hat{\Gamma }}_{a}=0.7$ predicts a range of 35–48 days for all five EVOOs. The calculation of ${\hat{\Gamma }}_{a}$ requires the availability of an unaged reference oil, which is onerous for some applications but can be useful in research. It is considered unlikely that criterion 4 is attainable without normalization, given the heterogeneity of EVOO cultivars.

Anselmi et al. used an RIR, $\Gamma_{1}=\nu_{3}:\nu_{4}$, to predict β-carotene concentration in EVOO. β-carotene concentrations of unaged oils were determined chemometrically, and the indicator proved to be a useful predictor ($R^{2}=0.71$) [25]. However, this ratio was investigated for the current study and found to not satisfy Criterion 2. Anselmi et al. also successfully used the ratio $\Gamma_{2}=\nu_{2}:\nu_{1270}$ as a predictor of the ratio of Lutein to β-carotene (L/β), which is an indication of olive fly infestation. However, not only does this mechanism not apply to the current study, but the intensity ratio is also very unstable due to the low intensity of the 1270 cm${}^{-1}$ peak compared to that from the 532 nm RRS excitation used by Anselmi et al. Another RIR is offered by Qiu et al. $\Gamma_{3}=\nu_{1}:\nu_{1655}$ to predict the FFA content of olive oil [10].

The indicators $\Gamma_{a}$ and $\Gamma_{b}$ were defined manually, but more powerful techniques might be used to formulate this, such as an absolute indicator. The principle component analysis of Raman spectra has been used with success for identifying oils [33] and might be a useful technique here. Otherwise, the 1D neural networks used previously for feature extraction from the fluorescence spectra of EVOO might be applied [34,35].

## 6. Conclusions

RRS was applied to extra virgin olive oil in order to estimate carotenoid concentration. In order to choose an ideal excitation wavelength, the fluorescence of EVOO was measured, but enumerating the RREP of carotenoids in oil would also be useful. An indicator is desired that is proportional to carotenoid concentration, accumulated oxidation, or the time aged. Four criteria were set to evaluate the predictive power of the indicators and were applied to the two Raman intensity ratios recommended herein, where $\Gamma_{a}$ satisfied at least the first three criteria. The fourth criterion might be potentially satisfied by using chemometric (e.g., HPLC) reference measurements, which were not available in this study. ${\hat{\Gamma }}_{a}$ satisfies all four criteria but requires a reference measurement from un-aged oil. When measurements from an un-aged reference oil are available, ${\hat{\Gamma }}_{a}$ provides a powerful predictor for EVOO age and accumulated oxidation.

## Figures and Tables

**Table 1:** Fitted Raman peaks in order of prominence (relative intensity).

Shift [cm^−1^]	Name ^1^	Emitter
1522	$\nu_{1}$	Carotenoids
1152	$\nu_{2}$	Carotenoids
1001	$\nu_{3}$	Carotenoids
1186		Carotenoids
1443		Fatty Acids
960	$\nu_{4}$	Carotenoids

^1^ Common names in the literature.

## Data Availability

The data presented in this study are openly available in Mendeley Data at https://doi.org/10.17632/45v8n4n68c.1 (accessed on 17 July 2023).

## Abbreviations

The following abbreviations are used in this manuscript:

CCD	Charge-Coupled Device
EEM	Excitation-Emission Matrix
(E)VOO	(Extra) Virgin Olive Oil
(F)FA	(Free) Fatty Acid
FWHM	Full Width at Half Maximum
FT-IRS	Fourier Transform Infrared Spectroscopy
FT-RS	Fourier Transform Raman Spectroscopy
HPLC	High-Performance Liquid Chromatography
IOC	International Olive Council
NIRRS	Near-Infrared Raman Spectroscopy
RIR	Raman Intensity Ratio
RS	Raman Spectroscopy
RRS	Resonant Raman Spectroscopy
RREP	Resonance Raman Excitation Profile
SERS	Surface Enhanced Raman Spectroscopy
SNR	Signal-to-Noise Ratio
UV	Ultraviolet
